# An ecological perspective on water shedding from leaves

**DOI:** 10.1093/jxb/erab479

**Published:** 2021-11-02

**Authors:** Anne-Kristin Lenz, Ulrike Bauer, Graeme D Ruxton

**Affiliations:** 1 School of Biological Sciences, University of Bristol, 24 Tyndall Avenue, Bristol, UK; 2 School of Biology, University of St Andrews, Dryers Brae, Greenside Place, St Andrews, UK; 3 McGill University, Canada

**Keywords:** Drip tips, drop impact, epicuticular wax, leaf inclination angle, leaf movement, leaf trait adaptation, splash erosion, trichomes, water repellency, water shedding

## Abstract

Water shedding from leaves is a complex process depending on multiple leaf traits interacting with rain, wind, and air humidity, and with the entire plant and surrounding vegetation. Here, we synthesize current knowledge of the physics of water shedding with implications for plant physiology and ecology. We argue that the drop retention angle is a more meaningful parameter to characterize the water-shedding capacity of leaves than the commonly measured static contact angle. The understanding of the mechanics of water shedding is largely derived from laboratory experiments on artificial rather than natural surfaces, often on individual aspects such as surface wettability or drop impacts. In contrast, field studies attempting to identify the adaptive value of leaf traits linked to water shedding are largely correlative in nature, with inconclusive results. We make a strong case for taking the hypothesis-driven experimental approach of biomechanical laboratory studies into a real-world field setting to gain a comprehensive understanding of leaf water shedding in a whole-plant ecological and evolutionary context.

## Teaser

The complex interaction between rain, vegetation, soil, and climate can only be understood by studying form–function relationships of leaves in a meaningful ecological context.

## Introduction

In times of global change, with increasing frequency of high-intensity rainfall events (Allan, 2011) aggravating the loss of arable soil through erosion while a growing world population demands ever more food (Pimentel *et al.*, 1995), the interaction of vegetation with atmospheric water has never been a hotter topic in plant science. Plants, and their leaves in particular, intercept rainfall and act as condensation surfaces for fog and dew, and thereby completely change how this precipitation reaches and impacts the ground below (Dunkerley, 2020). The retention of surface water on leaves not only alters the hydrological cycle by increasing evaporation, it also has diverse impacts on the plant itself (summarized in Dawson and Goldsmith, 2018).

The effects of surface water retention on plants are, however, strongly context dependent. On the one hand, persistent wetness on leaves can impede transpiration and photosynthesis (Aparecido *et al.*, 2016, 2017; Berry and Goldsmith, 2020); however, on the other hand, foliar water uptake (Berry *et al.*, 2019; Schreel *et al.*, 2020) can boost photosynthesis and growth (Eller *et al.*, 2013; Carmichael *et al.*, 2020). Depending on the relative solute content of the interstitial fluid and the surface water, nutrients can leach from the leaf (Tukey, 1970) or be taken up (Templer *et al.*, 2015). In epiphytic bromeliads, wettable leaves have gained an important function for water and nutrient uptake (Zambrano *et al.*, 2019), and many species store rain water in tightly sealed leaf ‘tanks’ (Freschi *et al.*, 2010; Ladino *et al.*, 2019). Leaf surface wetness has been shown to promote epiphyll and pathogen growth (Huber and Gillespie, 1992), but mutualistic fungi benefit too (Arnold *et al.*, 2003).

In general, it appears that temporary water cover on leaf surfaces can have benefits, but long-term wetness tends to be disadvantageous. Therefore, plants have ubiquitously evolved adaptations to promote water shedding from their leaves. These can be simplified into two general mechanisms: (i) increased water repellency of the leaf surface and (ii) steeper leaf inclination angle. In the following, we will explore both strategies in detail and discuss their interaction with each other as well as trade-offs with other leaf functions, and implications for leaf ecology and evolution. We will first consider the (simpler) case of a droplet or water layer on a static leaf, as might occur after rain or as a result of condensation, before exploring the more complex effects of drop impacts during rain. Finally, we will take a look at some specialized adaptations such as the ‘drip tips’ on the leaves of a diversity of tropical species, and anisotropic surface structures promoting directional water transport.

### Wetting versus water shedding

**Fig. 1.**
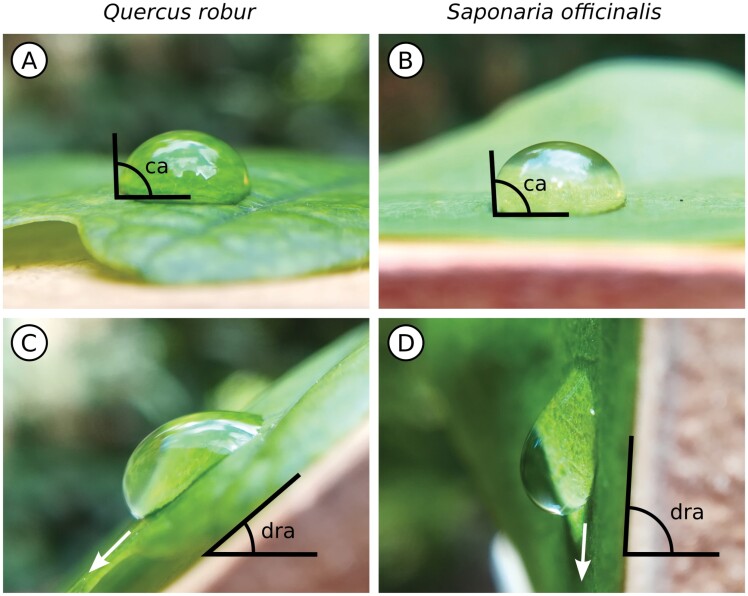
Two parameters that are commonly measured to characterize the interaction of water with leaf surfaces are the static contact angle of a sessile drop (A, B) and the drop retention angle of a sliding drop (C, D). The drop retention angle is the angle of a tilting stage at which a drop begins to slide or roll off. It is the more meaningful parameter in the context of water shedding, as can be seen by comparing static contact angles (ca) and drop retention angles (dra) on oak (*Quercus robur*, A, C) and soapwort (*Saponaria officinalis*, B, D) leaves. While the static contact angles on both leaves are very similar, the drop retention angles differ drastically.

**Fig. 2.**
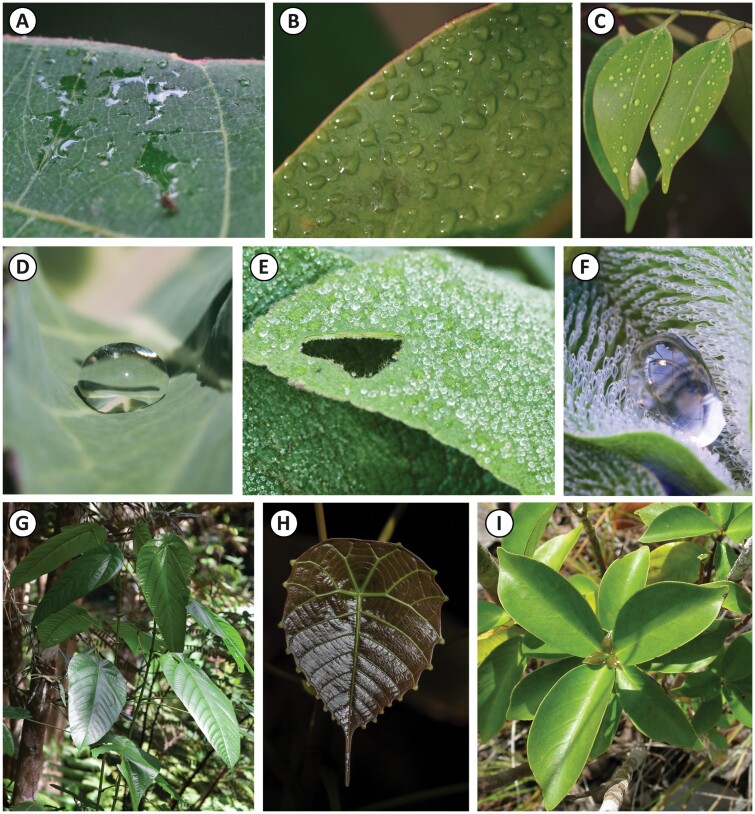
Leaf traits that affect water shedding. (A–C) Depending on the surface chemistry, leaves with relatively smooth surfaces can be either more (A, B) or less (C) wettable. (D–F) Three-dimensional surface features such as microscopic epicuticular wax crystals on glaucous leaves (D) or trichomes (E, F) act as enhancers of the intrinsic surface chemistry; in this case, they render the surfaces even more hydrophobic. (F) A special case is the so-called *Salvinia* effect, where hydrophobic trichomes with hydrophilic tips ‘pin’ a drop in place, away from the cuticular surface (Barthlott *et al.*, 2010). (G) Leaves on the same plant can be held at vastly different inclination angles. (H) Elongated apical drip tips are commonly found on the leaves of tropical rainforest understorey plants; here in Brunei, Northern Borneo. (I) Drip tips are absent from the leaves in a nearby open, dry habitat in Brunei, Northern Borneo.

Two factors dictate how easily water is shed from a leaf: (i) the water repellency and (ii) the inclination of the surface. In brief, the more easily a drop can move and the steeper the surface, the more easily water will be shed. Both factors are intuitively captured in the drop retention angle—the angle at which a drop starts to roll off a surface when the surface is gradually tilted (Fig. 1C, D). Note that we use ‘water repellency’ to describe the ease of drop movement across the surface. As we will show below, this is not synonymous with ‘non-wettability’ or hydrophobicity (Callies and Quéré, 2005), which is characterized by the contact angle of a sessile drop on the horizontal surface (Kung *et al.*, 2019; Fig. 1A, B). On hydrophilic surfaces, contact angles are small and drops spread; on hydrophobic surfaces, contact angles are large and drops are increasingly spherical in shape (Fig. 2A–D). The exact boundary between hydrophilic and hydrophobic has been disputed repeatedly (Guo *et al.*, 2008; Law, 2015). The maximum contact angle of water measured on a flat surface is 119° (Nishino *et al.*, 1999). Higher apparent contact angles are measured on rough surfaces where microscopic roughness can effectively act as an enhancer of the inherent chemical surface properties (Bico *et al.*, 2002; Bhushan and Jung, 2008). In extreme cases, contact angles of 180° (i.e. perfectly spherical drops) can be achieved (Herminghaus, 2000), most famously on the lotus leaf (Barthlott and Neinhuis, 1997).

As natural leaves are never perfectly flat and smooth, their wettability is always based on a combination of surface chemistry and topography (Koch *et al.*, 2008; Rosado and Holder, 2013). Surface chemistry is determined by the composition of the epicuticular wax layer that forms the major transpiration barrier on the epidermis of terrestrial plants and therefore tends to be hydrophobic (Jetter *et al.*, 2006); however, some leaf surfaces have been shown to be extremely hydrophilic (Bohn and Federle, 2004; Koch *et al.*, 2009). Papillae, trichomes, cuticular folds, and epicuticular wax crystals generally increase the surface roughness and may thereby influence the wettability (Wenzel, 1936; Koch *et al.*, 2008; Fig. 2D–F). Depending on their density and inherent wettability, trichomes have been shown to either impede (Brewer *et al.*, 1991) or enhance wetting (Bauer *et al.*, 2013; Pina *et al.*, 2016; Schreel *et al.*, 2020). Similarly, the effect of surface roughness is not straightforward, as becomes obvious when comparing the ‘petal effect’ (Feng *et al.*, 2008; Janairo *et al.*, 2016) and the ‘lotus effect’ (Barthlott and Neinhuis, 1997; Schulte *et al.*, 2011). In both cases, hierarchical surface structures result in apparent contact angles >150°, but depending on the aspect ratio, namely the height-to-distance ratio of the asperities, drops either stick or bead off (Bhushan and Jung, 2008; Lin and Chou, 2014; Gong *et al.*, 2015).

Traditionally, most studies on biological surfaces report static contact angles; however, the above examples illustrate that this may not be the best variable to explain water shedding from leaves. This is further aggravated because biological surfaces tend to be inhomogeneous in texture and chemistry (Barthlott *et al.*, 2010; Fig. 2F). Even on artificial surfaces, contact angles are poorly reproducible, with variation of up to 20° between repeated measurements (Gao and McCarthy, 2006). Empirical studies so far failed to show a consistent correlation between static contact angles and drop retention angles—the direct measure of how easily water sheds from a leaf (Brewer and Nuñez, 2007; Aryal and Neuner, 2010; Ginebra-Solanellas *et al.*, 2020; Fig. 1). This may not be too surprising because the dynamic contact angles of a sliding or rolling drop are often vastly different from the static contact angle on the same surface. Therefore, it is increasingly acknowledged that the more meaningful parameter for the understanding of water shedding from leaves is the contact angle hysteresis, namely the difference between the advancing and receding contact angle of a sliding drop (Brewer and Nuñez, 2007; Aryal and Neuner, 2010; Rosado and Holder, 2013). The lower the contact angle hysteresis, the lower the drop retention angle and the more easily the drop is shed from the surface. Water will run off if the leaf inclination angle exceeds the drop retention angle (Konrad *et al.*, 2012). A complicating factor is the drop size, especially for smaller drops on more hydrophilic leaves. As the drop rolls off, a thin water film will stay behind. This can eventually reduce the drop to a size where it stops to move (Oqielat *et al.*, 2011). Barfield *et al.* (1973) found that the amount of water retained on lettuce, tomato, and cucumber leaves was dependent on the drop size.

### Falling drops: bouncing, splashing, and impact wetting

**Fig. 3.**
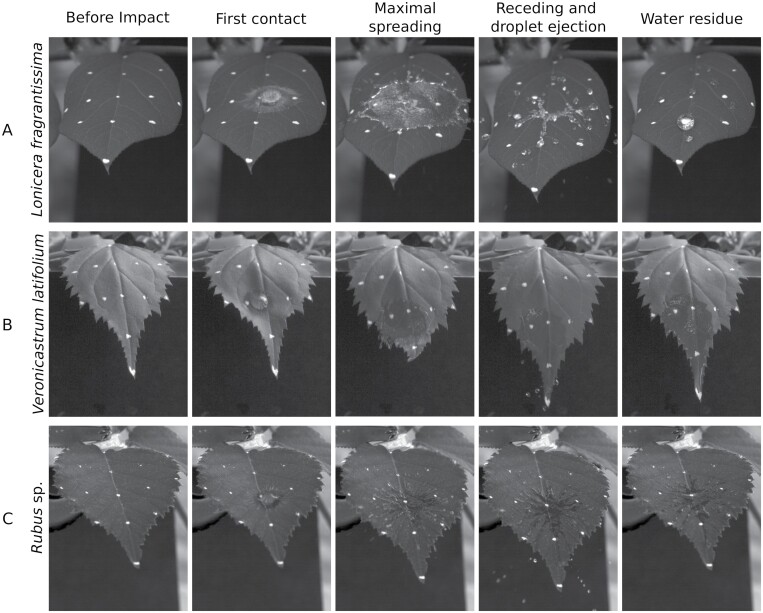
Impact behaviour of a water drop (volume=50 µl, speed ~3ms^–1^) on three different leaves. (A) Leaf surface hydrophobic (winter honeysuckle, *Lonicera fragrantissima*). The drop splashes and breaks up into smaller droplets which bounce off the surface. Some drops are decelerated enough to adhere to the surface. (B) Leaf surface hydrophilic (Chinese veil, *Veronicastrum latifolium*). The drop spreads and partly runs off as the leaf is tilted downward following the impact. The water residue is visible as a continuous wet area. (C) Leaf surface with trichomes (bramble, *Rubus* sp.). The drop spreads and breaks up irregularly (‘fingering’).

So far, we have considered the relatively simple case of a sessile drop on a static leaf. However, rain drops hit the leaf with impact forces of >1000 times their static mass (Soto *et al.*, 2014). Upon impact, drops either adhere, bounce, or splash, depending on a complex interplay of leaf surface wettability, leaf inclination angle, and the rigidity of the leaf and petiole, as well as drop size and impact velocity (Fig. 3). Recent studies have tried to disentangle these effects to a certain degree, but much remains yet to be understood. On horizontal leaf surfaces, drops up to a critical size and impact velocity tend to adhere, while larger and faster drops bounce or splash (Bassette and Brussière, 2008; Kwon *et al.*, 2014; Fig. 3A). The critical drop size and speed at which this transition occurs is 50–85% lower for hydrophobic than for hydrophilic leaves. Bouncing appears to be confined to hydrophobic leaves, where it occurs at intermediate drop sizes and impact velocities; larger and faster drops splash (Dorr *et al.*, 2015). Studies on artificial surfaces suggest that in cases where hydrophobicity is based on surface roughness, drop impacts above a critical velocity can enforce wetting, if the kinetic energy of the drop exceeds the energetic barrier for the water to penetrate in between the surface asperities (Bartolo *et al.*, 2006; Marengo *et al.*, 2011). This means that on hydrophobic leaves, raindrops with a low impacting speed will most probably roll or bounce off the surface, while heavy rainfall could lead to complete wetting of the leaves; however, this has not yet been investigated with natural leaf surfaces.

We already showed for the static case that the inclination angle determines the transition between drop retention and run-off for a surface of a given water repellency. For the dynamic case, it has been shown that the wettability strongly influences the transition points between adhesion, bouncing, and splashing. For hydrophobic leaves, higher inclination angles broaden the range of drop sizes and impact velocities at which bouncing occurs; that is, the steeper the leaf inclination, the less likely the drop will stick or splash (Kwon *et al.*, 2014; Dorr *et al.*, 2015). A similar shift is observed for hydrophilic leaves, but instead of bouncing, drops spread and run off (Bassette and Brussière, 2008; Fig. 3B). While these are generally valid trends, the drop behaviour on natural leaves depends on many more factors (e.g. the rigidity of the leaf as well as macro-topographic features such as vascular bundles or trichomes, Fig. 3C), and can differ drastically from the theoretical predictions (Papierowska *et al.*, 2019). Note that all of the above studies classify leaves by wettability (i.e. static contact angle) and not water retention. It is also worth noting that so far, studies have only considered individual drop impacts on previously dry leaves while, during natural rainfall, multiple drops will impact simultaneously or in short succession, and will interact with water already on the leaf surface. Interactions of multiple drops have so far mainly been explored theoretically (Rein, 1996; Damak *et al.*, 2016; Wang and Bourouiba, 2018). Lejeune and Gilet (2019) investigated the interaction of an impacting with a sessile drop on a Perspex surface experimentally, and found that both drops splash off the surface as one, with the splashing distance depending on the inclination angle of the surface.

### The effect of leaf shape

Narrow elongated leaves and extensions to the leaf apex—so-called ‘drip tips’ (Jungner, 1891; Fig. 2H)—are commonly interpreted as adaptations for improved surface water drainage. Because narrow elongated leaves also tend to have more pronounced drip tips, it is impossible to fully separate the effects of both. Numerous manipulative experiments on natural leaves (Dean and Smith, 1978; Williamson, 1981; Lightbody, 1985; Burd, 2007) and, more recently, observations on artificial leaves with and without drip tips (Lücking and Bernecker-Lücking, 2005; Wang *et al.*, 2020) all indicate that drip tips promote water shedding from the apex. By funnelling the surface water onto a narrow protrusion, drip tips increase the frequency but decrease the size of the shed drops (Lightbody, 1985; Lücking and Bernecker-Lücking, 2005). However, it is less clear how drip tips affect the overall rate of water removal. Dean and Smith (1978) and Ivey and De Silva (2001) both report that experimental removal of drip tips slowed down the drainage of surface water from leaves; however, Lücking and Bernecker-Lücking (2005) found that drip tips mainly reduced the drying time for the leaf apex, but not the entire lamina. It appears likely that drip tips on their own, without further adaptations of leaf geometry, inclination angle, and surface topography promoting water flow across the lamina towards the tip, are insufficient to have a significant effect on water shedding and drying times.

### Leaf movement

Up to here, we have ignored the fact that leaves can move. This includes both slow deformations as a result of loading, and rapid impact responses on a subsecond time scale. Upon impact, rain drops transfer momentum to the leaf, leading to a deformation of the leaf itself and/or the supporting petiole. The transfer of energy depends on the impact rate and location, the size and speed of the drop (which is influenced by wind and surrounding vegetation), and whether the drop bounces or spreads on the leaf. Drop behaviour on the surface in turn depends on drop size, speed, and impact angle, and on the leaf’s surface properties, size, rigidity, and angle of inclination, as well as presence or absence of water already on the surface. If part of the impacting water remains on the surface, the added mass will change the inclination angle, with the amount of change depending on the biomechanical properties of the leaf (Holder *et al.*, 2020). It should be clear from these general arguments that leaf movement is a particularly complex issue and, apart from the trivial point that impact-induced deflection will result in a temporary increase of leaf inclination angle and thereby aid water shedding, obtaining general trends remains difficult.

In laboratory experiments, leaves that were fixed at the base of the petiole responded to a drop impact with characteristic damped oscillations (Ginebra-Solanellas *et al.*, 2020; Holder *et al.*, 2020). Several studies showed the amplitude of these oscillations to be directly proportional to the transfer of momentum. However, momentum transfer did not simply scale with drop size, but was dependent on the impact location (Bauer *et al.*, 2015), material properties of the leaf (Ginebra-Solanellas *et al.*, 2020), and the wettability of its surface (Gart *et al.*, 2015). Using standardized beams of different surface properties and lengths, the latter study found not only higher impact transfer, but also a much stronger effect of the lever arm length for wettable compared with non-wettable surfaces. This is further complicated if the rigidity changes along the length of the leaf. Bhosale *et al.* (2020) found a ‘sweet spot’ around three-quarters along the length of *Katsura* sp. leaves, with more distal impacts leading to more bending of the leaf tip and less energy transfer to the whole leaf. In contrast to human-made materials, leaves are highly heterogeneous with a soft inner mesophyll encased in a layer of much stiffer epidermal cells (Onoda *et al.*, 2015). A scaffold of lignified and sclerified veins provides further rigidity, and the density and spatial organization of these veins is a major determinant of the deformation characteristics of the leaf (Roth-Nebelsick *et al.*, 2001).

**Fig. 4.**
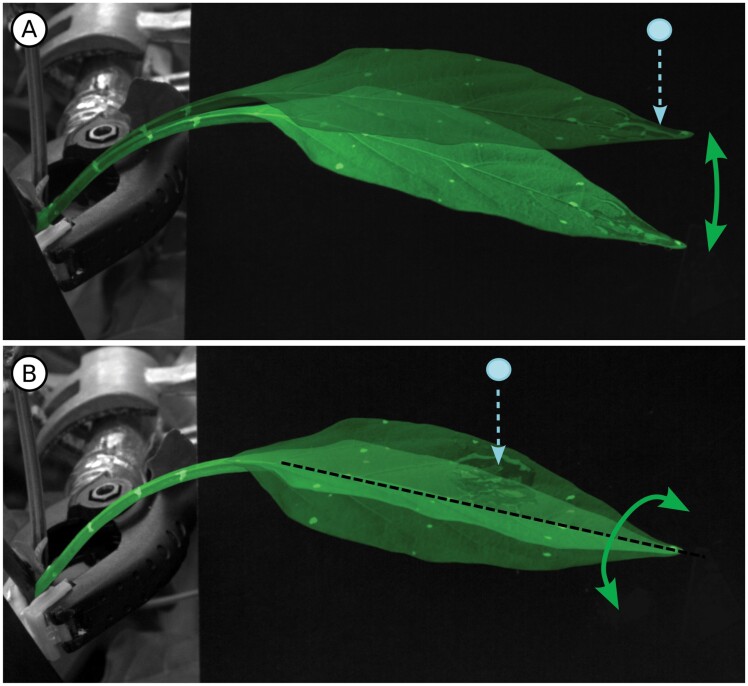
The drop impact response of a *Capsicum* sp. leaf is strongly dependent on the impact location. (A) An impact near the leaf tip leads to bending in the petiole and lamina. (B) A lateral impact mainly causes twisting in the petiole.

Apart from bending (Fig. 4A), leaves can also twist (Fig. 4B) or exhibit more complex movements such as undulating or flapping. Vogel (1992) defined the twist-to-bend ratio as the ratio of flexural rigidity to torsional rigidity, in order to categorize leaf movements due to petiole bending and twisting. Petioles with a non-circular cross-section favour twisting; however, the actual impact response is strongly dependent on the impact location (Fig. 4). Drops impacting along the midrib of superhydrophobic *Katsura* sp. leaves predominantly caused bending, while more lateral impacts increasingly caused twisting (Bhosale *et al.*, 2020). Petioles are composite structures with variable proportions of soft central parenchyma and supporting peripheral collenchyma which may be reinforced by sclerenchyma. It is worth noting that fully hydrated plant tissue consists of up to 98% water, and leaf and petiole stiffness is therefore crucially dependent on the hydration status (Niklas and O’Rourke, 1987; Niklas, 1999). Leaf movement is also likely to be more complex during natural rainfall, but the effect of multiple simultaneous or consecutive drop impacts remains uninvestigated to date.

### Towards a comprehensive understanding of water shedding

In order to understand water shedding in nature, we need to take into account that leaf shape, surface topography, inclination angle, and leaf movement all interact with each other, with rain characteristics such as drop size and frequency, with presence of water on the leaf surface, and with other factors such as wind. Few studies have looked at more than one or two of these factors in combination, and none so far has investigated the interaction of natural rainfall with leaves directly. A number of studies compared leaf inclination and drop retention angles, and found that natural leaf inclination angles mostly exceeded drop retention angles or, occasionally, were roughly equal. In the latter case, a temporary slight increase in inclination angle caused by a drop impact or added water load was sufficient to initiate water shedding (Holder, 2012; Ginebra-Solanellas *et al.*, 2020). Dean and Smith (1978) and Ellenberg (1985) investigated the combined effects of leaf inclination angle and drip tips. Both concluded that the inclination angle was the more influential of the two factors. Nanko *et al.* (2013) investigated the combined effect of leaf inclination angle and surface properties, and found that the maximal size of drops shed from the leaves decreased with increasing inclination angle and hydrophobicity.

In the context of leaf inclination angle, it is particularly interesting to consider topographic features that promote directional water transport across the leaf surface. For example, longitudinal cuticular ridges on rice (*Oryza sativa*) leaves favour water movement along the leaf blade (Kwon *et al.*, 2014), and unidirectionally angled trichomes on ryegrass (*Lolium* sp.) leaves result in a 3-fold lower drop retention angle in the proximal than in the distal direction along the leaf (Guo *et al.*, 2012). Hierarchical groove structures and overlapping acute-angled arches on the hydrophilic trap rim of carnivorous *Nepenthes* pitcher plants cause highly effective water transport in the outward direction (Chen *et al.*, 2016) and may play a role in preventing the dilution of the trap fluid with rain water during tropical downpours.

How can we predict leaf deformation due to surface water and movement in response to rain impact? Tadrist *et al.* (2014) modelled how leaf angles change under wind and static loading depending on various leaf traits. For static loading, they defined the elasto-gravity number which combines the relative lengths of petiole and lamina with the flexural rigidity of the petiole and the mass of the lamina. The higher the elasto-gravity number, the more easily the leaf will bend down and shed water. Under wind loading, the size of the lamina and the torsional rigidity of the petiole determine the amount of water shedding due to tilting. Vogel (1989) showed that the large, lobed leaves of the tulip tree (*Liriodendron tulipifera*) can curl up under wind load, thereby reducing drag and fluttering. This not only reduces the total exposed surface area but also mainly exposes the more hydrophobic abaxial surface (Goldsmith *et al.*, 2017). On the other hand, fluttering and increased surface exposure to wind should promote water shedding and evaporation. Therefore, leaf curling might be counterproductive from a water shedding perspective and is more likely to be an adaptation for the prevention of damage. Further progress on understanding the complex interplay between leaves, rain, and wind requires a move away from laboratory studies to situations where leaves are held naturally and are subject to natural rainfall or condensation. The increasing availability of small, lightweight, and weatherproof measurement devices, affordable and powerful high-speed video cameras, and low-cost microcomputers for automated data collection make this approach more feasible than ever before (Chan *et al.*, 2016; Kwok, 2017; Duke *et al.*, 2019; Oellermann *et al.*, 2021, Preprint).

### Implications of water shedding for soil erosion

One interesting line of argument proposes that a major driver behind the evolution of leaf shape and surface properties might be to reduce the size of shed drops, and thereby mitigate splash erosion and nutrient loss in the immediate vicinity of the plant. As discussed before, smaller drops reach lower terminal velocity and therefore transfer less momentum upon impact. Interception by leaves has been shown to reduce average throughfall drop size by half in comparison with unfoliated trees (Nanko *et al.*, 2016). Foot and Morgan (2005) looked at the effect of leaf inclination angles on the soil particles in a lab experiment. They found no difference between soil particle detachment below three different plant species and on bare soil, and concluded that the effects of leaf drips and shielding through the canopy probably cancel each other out. Nanko *et al.* (2013) compared the drop sizes shed from needles and broadleaves of different hydrophobicity. For broadleaves, drop size decreased with water repellency of the leaves. For needles, drop size was inconsistent and strongly depended on needle arrangement on the branch. Drops were frequently pinned between the tips of multiple needles, resulting in very large drops eventually being shed.

Drip tips also reduce the size of intercepted rain drops falling from leaves and thereby the impact energy transferred to the ground (Williamson, 1981; Lücking and Bernecker-Lücking, 2005; Burd, 2007). Williamson (1981) suggested testing this hypothesis by looking at the prevalence of drip tips in relation to soil type. If erosion was a major selection factor, drip tips should be less common in sites that are less prone to splash erosion, such as in swamps or on sandy soils. In contrast, there should be no such effect if the key selection pressure was the removal of water from the leaf surface. Williamson (1981) provides anecdotal evidence that drip tips are absent from mangrove forests, but a systematic survey of leaf shapes in regularly water-inundated habitats has not yet been done. In contradiction to the erosion hypothesis, Rebelo and Williamson (1996) found a higher prevalence of drip tips in two areas of Amazonian rainforest with sandy soil than in a nearby site with clay; however, this study is limited to a few individual sites and does not consider that in tropical rainforests, the forest is commonly covered in leaf litter.

Williamson *et al.* (1983) also investigated the height distribution of drip tips in lowland rainforest, arguing that absence of drip tips from foliage very close to the ground—at <50cm height—could also be interpreted as supportive of the erosion hypothesis because drops falling from such a low height have little kinetic energy. Conversely, water removal as a key selection factor should lead to higher prevalence of drip tips close to the ground where evaporation is hindered by high air humidity. In line with the erosion hypothesis, drip tips were less prominent closer to the ground (Williamson *et al.*, 1983); however, the detailed relationship between leaf height on the one hand, and drop velocity and energy transfer to the substrate on the other hand, remains unstudied to date. While the erosion hypothesis is certainly intriguing, we feel that there is insufficient experimental evidence to draw evolutionary conclusions at present.

### Trade-offs and synergies with other leaf functions

**Fig. 5.**
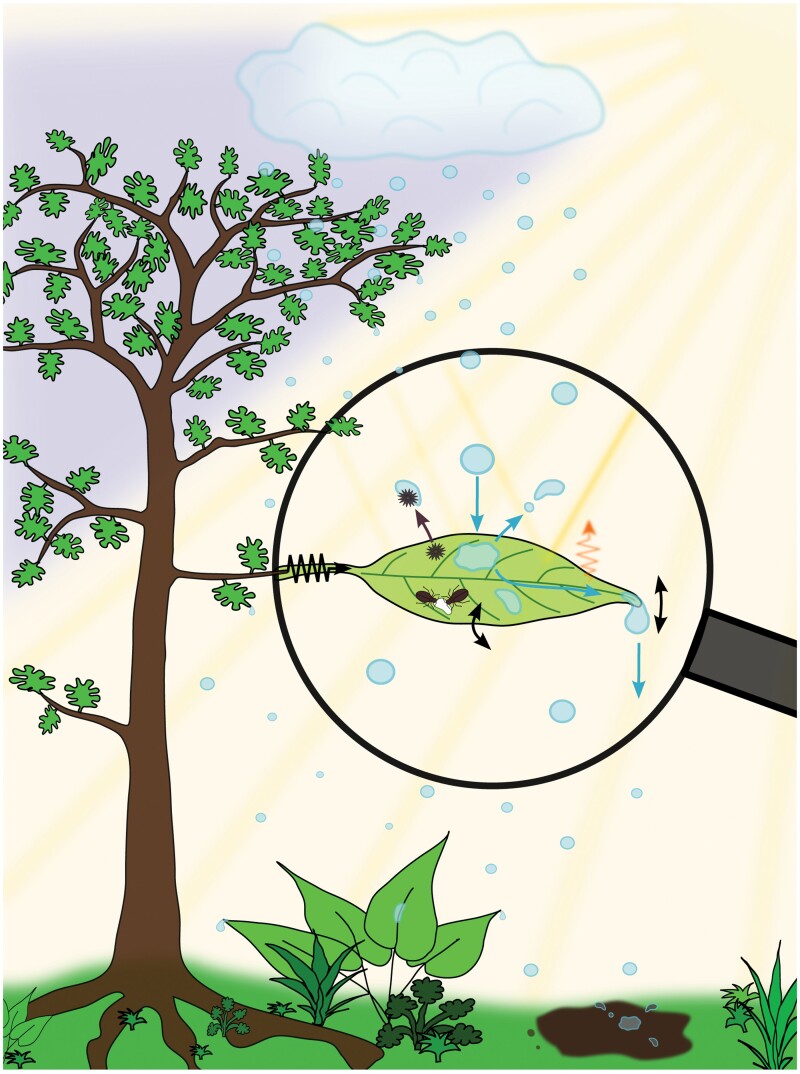
An integrated view on water shedding in the greater ecological context. Leaves are multifunctional organs that need to balance photosynthesis, hydration, nutrient demand, and temperature control with resistance to mechanical stress, herbivores, and pathogens. Water shedding is further influenced by the source of leaf surface water (different types of rain, throughfall, drip water from other leaves, mist, and condensation), the position of the leaf on the plant and within the surrounding vegetation, the biomechanical properties of the entire plant, and additional weather factors such as wind. Water shed from leaves in turn impacts other leaves further down, or can cause splash erosion and nutrient loss upon impact on the soil.

Effective water shedding is only one of many functional demands on leaves and, as an evolutionary driver, it probably plays a minor role behind other selection factors that determine photosynthetic efficiency, construction costs, and life span, the combination of which defines the carbon economics of a given leaf (Donovan *et al.*, 2011; Fig. 5). Photosynthetic performance needs to balance water and light availability with gas exchange and temperature control. The selective power of this trade-off is well illustrated in the contrast between sun and shade leaves. Water on the surface can reduce water loss by transpiration and mitigate the risk of overheating; however, it also impedes light penetration and gas exchange, and the overall impact on photosynthesis tends to be negative (Aparecido *et al.*, 2017; Berry and Goldsmith, 2020). Photosynthetic performance, nutrient availability, exposure to mechanical stress, and effective defence against herbivores and pathogens together define the trade-off between structural investment and life span. Again, rain water impacting and accumulating on the leaf surface is a contributing factor as it can cause nutrient leaching (Tukey, 1970), impose mechanical stress, and provide a suitable environment for pathogenic bacteria and fungal spores (Huber and Gillespie, 1992). Moreover, rain drops impacting on already wet leaves and the resulting splashes can effectively distribute pathogenic spores to other nearby leaves (Gilet and Bourouiba, 2014; Kim *et al.*, 2019). In addition to physiological and ecological trade-offs, leaf size and shape are also subject to developmental constraints (Nicotra *et al.*, 2011). The evolution of leaf traits that promote water shedding therefore has to be viewed in the greater context of leaf function and development.

Plant surface features that may render leaves water repellent, such as trichomes and epicuticular wax crystals, will also affect light penetration, gas exchange, and temperature control (Riederer and Müller, 2006); however, the interdependence of these physiological processes makes it nearly impossible to quantify individual trade-offs. Dense trichome cover can reduce light penetration and photosynthetic performance significantly (Ehleringer *et al.*, 1976). A commonly cited adaptive function of epicuticular wax crystals is protection from harmful radiation by increasing UV reflectance (Reicosky and Hanover, 1978; Mauseth, 1988; Robinson and Osmond, 1994; Grant *et al.*, 2003). Bukhanov *et al.* (2019) reported UV-induced fluorescence in the epicuticular wax crystal layer of wheat (*Triticum* sp.). This could provide a mechanism to shift harmful UV radiation into the visible spectrum, thereby increasing light availability for photosynthesis. Synergy effects are likely between water repellency and protective functions against herbivore and pathogen attacks. Epicuticular wax crystals not only impede drop adhesion but equally prevent insects from attaching to the surface (Eigenbrode and Espelie, 1995; Gorb *et al.*, 2005). Water shedding from epicuticular waxes can help to remove debris and fungal spores from the surface (Barthlott and Neinhuis, 1997; Neinhuis and Barthlott, 1998). A similar effect has also been demonstrated for drip tips where increased water shedding prevented the accumulation of debris (Lücking and Bernecker-Lücking, 2005) and fungal spores on the leaf surface (Ivey and De Silva, 2001).

Leaf inclination angles undoubtedly affect water shedding; however, for leaves with water-repellent surfaces, a small deviation from the horizontal is sufficient. The large natural variation in leaf angles (Fig. 2G) is more convincingly explained by physiological demands such as optimized light capture, photoprotection during times of high light intensity, and temperature control (King, 1997; Falster and Westoby, 2003). Thus, the existence of traits that enhance water shedding from leaves does not imply that water shedding is an important aspect of the selective regime that has led to the expression of these traits.

An alternative approach to testing the adaptive value of traits is to look at the ecological context in which these traits prevail. Comparisons of contact angles on leaves across habitats failed to show an association of hydrophobicity with wetter climate (Holder, 2007; Goldsmith *et al.*, 2017; Sikorska *et al.*, 2017). Instead, low wettability was characteristic of a cold and dry climate (Aryal and Neuner, 2010; Goldsmith *et al.*, 2017). Brewer and Nuñez (2007) proposed that frequent heavy rainfall might remove fragile epicuticular waxes from leaf surfaces and thus increase wettability. Experiments with simulated rain on a range of common crops provided some evidence for wax crystal erosion (Baker and Hunt, 1986). An alternative hypothesis is that low wettability is favoured in arid environments because it can help to channel any water falling or condensing on the leaves towards the soil (Holder, 2007; Masrahi, 2020). In this case, one would expect leaf surface topography, geometry, and inclination angles adapted to funnel surface water towards the stem, and thereby the root system of the plant.

A few studies provide direct evidence linking the prevalence of drip tips to wetter habitats (Lightbody, 1985; Schneider *et al.*, 2003; Fig. 2H, I). Meng *et al.* (2014) argued that steep inclination angles and drip tips may be alternative strategies for water shedding. Low light conditions in the forest understorey should favour drip tips because a steep leaf angle will further reduce light interception. Indeed, drip tips were more common and leaves more horizontal in shade-adapted than in sun-adapted species in the same rainforest site. The majority of studies, however, examined species distributions more broadly and used statistical models to correlate the presence of drip tips, determined from images or herbarium specimens, with large-scale climate variables. This approach is problematic not only because many of the investigated climate variables are closely correlated with each other, but also because it ignores small-scale temporal and spatial variations of the microclimate that the plant experiences, as well as intraspecific variability and developmental plasticity of the leaf morphology. Consequently, the results are highly inconsistent: drip tip occurrence was correlated with total precipitation during the wettest season in Amazonian rainforest (Malhado *et al.*, 2012), with annual temperature along an elevation gradient in Peru (Goldsmith *et al.*, 2017), and with none of the tested parameters in a South American mixed savannah–woodland (Sullivan and Queenborough, 2020). Fritsch *et al.* (2018) slightly improved the study design by comparing herbarium specimens with climate data from their respective place of collection, thereby taking regional variation into account. Rainfall during both the wettest and the driest quarter best predicted the extent of drip tips on North American redbud (*Cercis* sp.) leaves; however, both climate parameters were also intercorrelated, making further interpretations difficult. The authors suggest that drip tips might be selected against in drier climates where retaining nightly accumulating dew on the leaves may reduce transpirational water loss in the morning.

### Plasticity of leaf traits

We already saw that inferring functional or evolutionary relationships from the presence or absence of certain leaf traits in different (micro-) habitats can be problematic for various reasons. Maybe the most imminent problem for such survey-based studies is that virtually all of the traits we considered in the context of water shedding are highly variable not only between individuals of the same species, but also between leaves of the same individual, and even within the same leaf over time. Epicuticular wax crystals can be reduced or absent in plants growing under low light conditions (Hallam, 1970) or under elevated air humidity (Koch *et al.*, 2006). Seasonal changes of epicuticular wax load and composition are widespread and have been attributed to leaf ontogeny (Jetter and Schäffer, 2001), temperature and water availability (Ziv *et al.*, 1982; Jordan *et al.*, 1983), and erosion of wax crystals over time (Neinhuis and Barthlott, 1998; Kang *et al.*, 2018). Multiple studies report an increase in leaf wettability for broad-leaved trees towards the later part of the growth season, namely with increasing leaf age (Neinhuis and Barthlott, 1998; Tranquada and Erb, 2014; Kang *et al.*, 2018; Xiong *et al.*, 2018).

Drip tips are both more common and more pronounced in saplings than in mature trees of the same species (Leigh, 1975; Zhu, 1997; Panditharathna *et al.*, 2008). It remains unclear whether this is due to different physiological demands, developmental constraints, or differing height off the ground. The importance of relative leaf height is corroborated by multiple reports of drip tips being more widespread in understorey than in subcanopy species, and least common in canopy species (Rollet, 1990; Roth *et al.*, 1990; Yáñez-Espinosa, 2003). How drip tip formation is regulated during leaf development remains unstudied to date, leaving the amount of trait plasticity in response to short-term environmental variation open to speculation. Transplant experiments with species that show a high variation of drip tip prevalence throughout their distributional range could provide first insights into this interesting question.

By far the most plastic of the leaf traits considered in this review is leaf inclination angle which can be highly variable depending on leaf and plant age (Liu *et al.*, 2019) and position on the plant (Niinemets and Fleck, 2002; Fig. 2G). However, leaf inclination angles are not only variable in space—they can also change with the seasons (Raabe *et al.*, 2015) and even with the time of day (Dean and Smith, 1978; Lovelock *et al.*, 1994). Seasonal changes of leaf inclination angle have been most commonly linked to light intensity and angle of radiation (Falster and Westoby, 2003; Meng *et al.*, 2014), but also to temperature control and drought resistance (Ryel and Beyschlag, 1995; Gratani and Bombelli, 2000; Raabe *et al.*, 2015; Peguero-Pina *et al.*, 2020). Furthermore, the influence of water load or drop impacts causes a temporary increase of leaf inclination angles as discussed above.

Plants can also actively change leaf angles in response to touch stimuli (reviewed by Braam, 2005) and to the time of day (reviewed by Minorsky, 2019). Circadian rhythms of leaf movements have mostly been attributed to changes of light incidence (Lovelock *et al.*, 1994; Peguero-Pina *et al.*, 2020), but also to patterns of the gravimetric tide (Barlow, 2015). Although there is currently no evidence that rhythmic temporal changes of leaf inclination angle are related to water shedding, it is conceivable that, especially in humid tropical environments, steeper nocturnal leaf angles facilitate drainage of rain water and condensation from the leaf surface. Dean and Smith (1978) even suggested that the leaves of some tropical plants change to a more vertical inclination in direct response to rainfall. However, they did not provide any data to support this assertion. Active changes in leaf angle in response to drop impacts have only been shown for *Mimosa pudica* touch-me-not plants (Applewhite, 1972; Wallace *et al.*, 1987); however, the ecological relevance of this behaviour with regards to water shedding has not been investigated to date.

## Conclusions and outlook

Leaves certainly vary in their water-shedding properties, and this variation is linked to a number of leaf traits. What has not been established is whether these leaf traits evolved in part because of the effect they have on water shedding. We discussed a number of studies that correlated leaf traits with site-specific climate and soil variables in the context of water shedding. This work has been generally equivocal and does not provide evidence for a direct impact of leaf traits associated with water shedding on fitness correlates such as plant survival, growth, or seed production. While the available field studies suffer from too much reliance on multifactor correlations and too few mechanistic experiments, our functional understanding of water-shedding processes from leaves is almost entirely based on laboratory experiments involving artificial rain and surfaces. These studies have been valuable in helping to develop a theoretical framework; however, in order to further advance our understanding of water shedding, experiments will have to move out of the lab and into the field.

One problem with lab studies is that surface and material properties of leaves start changing as soon as a leaf is separated from the plant. Laboratory air is typically very dry, and contact angle measurements can strongly depend on relative air humidity (Gledhill *et al.*, 1977; Hołysz *et al.*, 2008). Impact responses of leaves depend not only on the properties of the leaf itself, but also on the structural properties of the petiole, and the branch or stem that the leaf is attached to. Lastly, the nature of water shedding from a leaf will be affected by the nature of the rainfall (e.g. duration, drop size, and drop frequency), the effects of other climatic variables such as wind and air humidity, the stature and structural rigidity of the entire plant, and even the surrounding vegetation (influencing whether drops impacting on a particular leaf are direct rainfall or previously shed from higher vegetation). Water will impact not only on leaves but also on all other above-ground parts of a plant. Much of the water impacting on lower leaves of a plant will have been shed from further up the plant, and much of that water will ultimately end up in the substrate around the roots of the plant.

Future experiments should combine hypothesis-driven manipulative approaches (e.g. transplant experiments and targeted manipulations of leaf traits that affect water shedding) with biomechanical measurements (e.g. accelerometry or 3D motion analysis of leaf impact responses) and quantification of whole-plant fitness correlates (e.g. growth, survival, or seed set) in a natural field setting. While this approach is certainly challenging, we have now, for the first time, the necessary technology to take research on water shedding to the next level (Bauer *et al.*, 2020). In order to really understand water shedding in nature, we have to consider the complex interplay of leaf and plant biomechanical properties within the greater framework of the natural environment.
